# Biocatalysis of ursolic acid by the fungus *Gliocladium roseum* CGMCC 3.3657 and resulting anti-HCV activity†

**DOI:** 10.1039/c8ra01217b

**Published:** 2018-05-03

**Authors:** Shaobin Fu, Qingfeng Meng, Junshan Yang, Jiajia Tu, Di-An Sun

**Affiliations:** Institute of Medical Plant Development, Chinese Academy of Medical Sciences, Peking Union Medical College Beijing 100193 China diansun@sina.com fushb@126.com +86-0851-28609493 +86-10-57833298 +86-0851-28642513; Pharmacy School of Zunyi Medical University Zunyi 563000 China; Department of Public Health, Zunyi Medical University Zunyi 563000 China

## Abstract

Biocatalysis of ursolic acid (UA 1) by *Gliocladium roseum* CGMCC 3.3657 was investigated. Baeyer–Villiger oxidation was found to occur during the reaction. Four metabolites were isolated from the cultures and their structures were identified as 21-oxo,A-homo-3*a*-oxa-urs-12-en-3-one-28-oic acid (2), 21-oxo-3,4-seco-ursan-4(23),12-dien-3,28-dioic acid (3), 21β-hydroxyl-A-homo-3*a*-oxa-urs-12-en-3-one-28-oic acid (4) and 21β-hydroxyl-3,4-seco-ursan-4(23),12-dien-3,28-dioic acid (5), based on their NMR and MS spectral data. All of the four metabolites were new and their anti-HCV activity was tested. Their biotransformation pathway was also proposed.

## Abstract 2

Biocatalysis of ursolic acid (UA 1) by *Gliocladium roseum* CGMCC 3.3657 was investigated.

## Graphical abstract

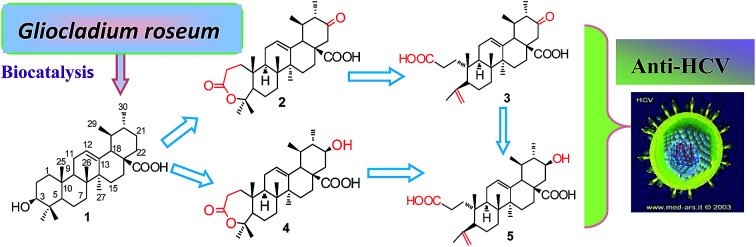

## Introduction

Ursolic acid (UA) is a pentacyclic triterpene acid capable of inhibiting various types of cancer cell. It was reported that UA inhibited several human breast cancer cell lines by activating caspase 3 and poly(ADP-ribose) polymerase cleavage, and this resulted in apoptosis.^1^ It can inhibit the cell proliferation of the human lung cancer cell line A549 by blocking cell cycle progression in the G1 phase which decreases the protein expression.^2^ Protein tyrosine phosphatase 1B (PTP1B) is a key element in the negative regulation of the insulin signalling pathway and may play an important role in diabetes and obesity.^3^ UA can induce apoptosis by activating p53 and caspase-3 gene expression and suppressing the NF-κB mediated activation of bcl-2 to inhibit B16F-10 melanoma cells.^2^ UA has also demonstrated antibacterial activity against strains *Enterococcus faecalis* and *Staphylococcus aureus*,^4^ and has an anti-arthritic,^5^ anxiolytic-like^6^ and hepatoprotective effect.^7^ Furthermore, UA exhibits potential anti-diabetic^8^ and obesity prevention effects.^9^

Structural modification of UA was carried out to improve its solubility and biological activity.^10–12^ Since UA does not have a lot of active sites for traditional organic modification, biocatalysis was investigated as a very good method to modify UA to obtain new metabolites.^13–15^

The hepatitis C virus (HCV) infects 170 million people around the world and is mostly transmitted *via* parenteral routes. The increased risk of HCV development in HCV-infected patients arises from the development of liver fibrosis and cirrhosis as a result of chronic inflammation.^16^ HCV entry inhibitors could satisfy a tandem mechanism for use with other inhibitors of viral replication, ultimately leading to a multifaceted approach for the eradication of the HCV infection. Recently, the inhibitory activity on HCV entry of EA and a series of derivatives was investigated, and EA and some of the derivatives showed potency for anti-HCV entry activity.^17–19^

This paper reports the microbial transformation of UA and also pentacyclic triterpene acid by filamentous fungus *G. roseum* CGMCC 3.3657. Hydroxyl and ketone groups were introduced in a regio- and stereo-selective way at C-21 which is difficult for organic synthesis. We suggest that ring A may be catalyzed by Baeyer–Villiger (BV) enzymes to form a seven-membered ring lactone and thus ring A can be cleaved. Compounds 2 and 3 exhibited better anti-HCV activity than 1, 4 and 5. This result indicates that the ketone group at C-21 may help to block HCV entry. Furthermore, the biotransformation pathway was also proposed.

## Results and discussion

### Identification of the products

**Fig. 1:**
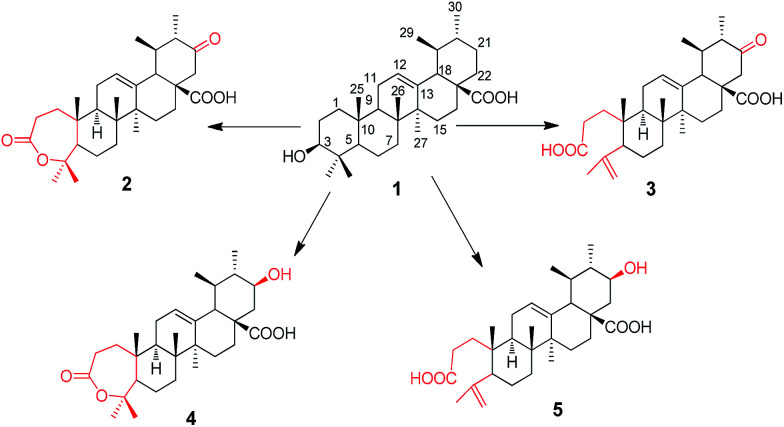
Microbial transformation of UA by *G. roseum* CGMCC 3.3657.

UA was incubated with the fungus *G. roseum* CGMCC 3.3657. Four transformation products were afforded (Fig. 1).

**Fig. 2:**
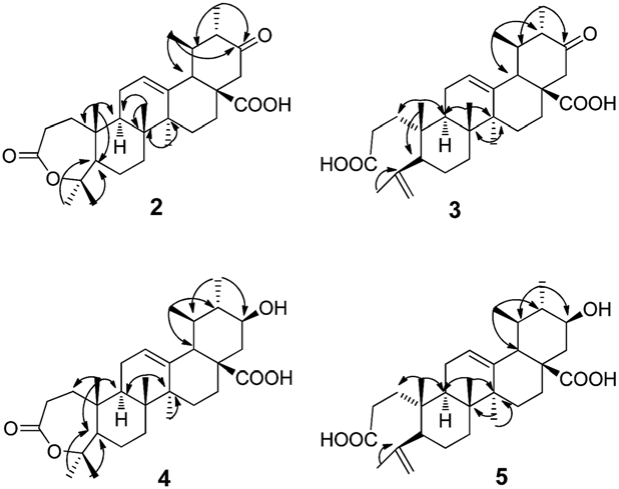
The key HMBC of compounds 2–5.

Metabolite 2 was more polar than the substrate. The HR-ESI-MS data ([M−H]^−^: 483.3104) of 2 (Appendix 2-1†) suggested a molecular formula of C_30_H_44_O_5_. There were two more oxygen atoms than the substrate (1). The ^1^H-NMR spectrum (Appendix 2-2 and 2-3†) revealed the presence of seven methyl groups, five with a singlet and two with a double peak. The signal at *δ*_H_ 3.46 assigned to H-3α in the substrate disappeared in product 2. The carbon signals (Appendix 2-4†) changed significantly, indicating the presence of two carboxyl groups (*δ*_C_ 174.2 and *δ*_C_ 175.7). Analysis of the HMBC data (Fig. 2) revealed that the carbonyl group (*δ*_C_ 209.5) was at C-21 based on the correlation between C-21 and H-22 (*δ*_H_ 2.65 and *δ*_H_ 2.39) and the correlation between C-21 and H-30 (*δ*_H_ 0.93, d and *J* = 6.6 Hz). The carboxyl group *δ*_C_ 174.2 was assigned to C-28 based on the HMBC data (Fig. 2) due to the correlation between C-28 and H-22. The signal at *δ*_C_ 85.4, correlated with both H-23 (*δ*_H_ 1.38) and H-24 (*δ*_H_ 1.33) in the HMBC spectrum (Fig. 2), may be attributed to C-3 or C-4 (Appendix 2-6 and 2-7†). This was further identified as C-4 and the signal at *δ*_C_ 175.4 was confirmed to be C-3 with reference to the data of a known compound (A-homo-3*a*-oxa-olean-12-en-3-one-28-oic acid),^20^ which is structurally similar to product 2. Based on the above evidence, product 2 was elucidated as 21-oxo,A-homo-3*a*-oxa-urs-12-en-3-one-28-oic acid.

Metabolite 3 showed an [M−H]^−^*m*/*z* peak at 483.3105 on the HR-ESI-MS spectrum (Appendix 3-1†), corresponding to the molecular formula of C_30_H_44_O_5_. In the ^1^H-NMR spectrum (Appendix 3-2†), two more olefinic proton signals at *δ*_H_ 4.65 and *δ*_H_ 4.86 with respective singlet peaks appeared besides H-12 at *δ*_H_ 5.40 (t, *J* = 3.6 Hz), and only six methyl group signals were observed. Correspondingly, the ^13^C-NMR spectrum (Appendix 3-3†) revealed two new olefinic carbon signals at *δ*_C_ 113.7 and *δ*_C_ 147.2. The protons at *δ*_H_ 4.65 and *δ*_H_ 4.86 were directly attached to the carbon at *δ*_C_ 113.7. Additional olefinic carbon signals at *δ*_C_ 127.6 and *δ*_C_ 137.2 were identified as C-12 and C-13, compatible with the substrate. This implied that compound 3 could be a product with ring-cleavage. From the HMBC data (Fig. 2 and Appendix 3-5 and 3-6†), HMQC data (Appendix 3-4†) and previous literature,^20,21^ we can conclude that ring-A was cleaved between C-3 and C-4, C-3 was oxygenated to become a carboxyl group and a new double bond was formed between C-23 and C-4. Like for metabolite 2, the carbon signal at *δ*_C_ 209.5 was a carbonyl group and assigned to C-21 based on the HMBC spectrum. Therefore, product 3 was confirmed as 21-oxo-3,4-seco-ursan-4(23),12-dien-3,28-dioic acid.

Metabolite 4 had the molecular formula of C_30_H_46_O_5_, as evidenced by the HR-ESI-MS data ([M−H]^−^: *m*/*z* 485.3263, shown in Appendix 4-1†). According to the ^1^H-NMR spectrum (Appendix 4-2†) and ^13^C-NMR spectrum (Appendix 4-3†), the structure of compound 4 was similar to that of compound 2. All of the methyl groups were unchanged compared to those of the substrate. In addition, ring-A also formed a seven-membered ring lactone. The double bond with a chemical shift of *δ*_C_ 126.4 and *δ*_C_ 137.5 occurred between C12 and C13 which was consistent with the substrate. The carbon signal at *δ*_C_ 71.2, which correlated with the proton signal at *δ*_H_ 3.43, was confirmed to be C-21 from the HMBC data (Fig. 2 and Appendix 4-5†) whereas C-21 was a carbonyl group in metabolite 2. Additionally, the orientation of the hydroxyl group at C-21 was deduced as the β-position from the coupling constants and splitting pattern of H-21 (*δ*_H_ 3.43, td, *J* = 4.2, 10.8 Hz) in the ^1^H-NMR spectrum which was at its axial (α) position. Ultimately, metabolite 4 was determined as 21β-hydroxyl-A-homo-3*a*-oxa-urs-12-en-3-one-28-oic acid.

The molecular formula of metabolite 5 was determined as C_30_H_46_O_5_ from its HR-ESI-MS spectrum ([M−H]^−^: 485.3215, shown in Appendix 5-1†). In the ^1^H-NMR spectrum (Appendix 5-2†), there were proton signals at *δ*_H_ 4.65 and *δ*_H_ 4.84 which were similar to those of compound 3. Correspondingly, the olefinic carbon signals at *δ* 113.4 and *δ* 147.1 in the ^13^C-NMR spectrum (Appendix 5-3†) were also consistent with metabolite 3. It was deduced that metabolite 5 was also a product with ring-A cleavage. The new double bond was assigned between C-4 and C-23, identical to compound 3. The two carboxyl groups were attributed to C-3 and C-28, with chemical shifts at 174.7 ppm and 177.4 ppm respectively. Moreover, the secondary hydroxyl group was assigned to C-21, based on the proton signal at *δ*_H_ 3.19 which correlated with the carbon signal at *δ*_C_ 69.0 in the HMQC spectrum (Appendix 5-4†). The orientation of the –OH group was determined to be its β-position from the coupling constants and splitting pattern of H-21 (td, *J* = 4.2, 10.8 Hz). Based on the above evidence, metabolite 5 was identified as 21β-hydroxyl-3,4-seco-ursan-4(23),12-dien-3,28-dioic acid.

### Anti-HCV activity of UA and biotransformation products

**Fig. 3:**
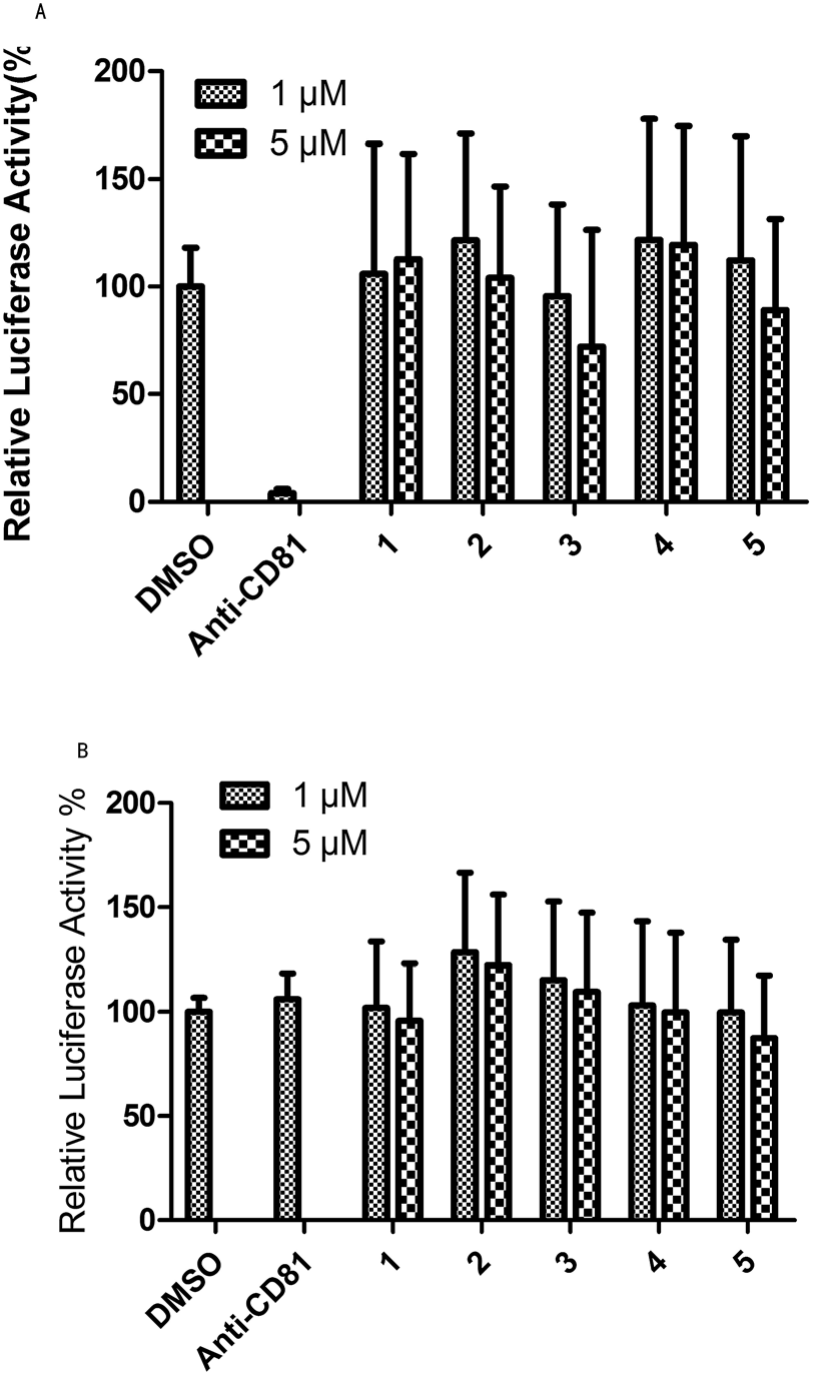
An anti-HCV activity test based on HCVpp and VSVG-pp entry assay: 1 is the substrate and 2–5 are products.

Evaluation of the anti-HCV activity of UA and its transformation products was achieved in this study for blocking HCV entry. The development of a HCV pseudo-particle (HCVpp) and vesicular stomatitis virus G protein pseudo-particle (VSVG-pp) has made it possible to readily observe HCVpp-mediated cell entry *in vitro*. Fig. 3A shows the results of the test for HCVpp entry blocking. Concentrations of 1 μM and 5 μM of the studied compounds and 1% DMSO, as the solvent control, were evaluated and anti-CD81 was used as a positive control which emulated the blocking of HCV entry. From Fig. 3, compound 3 could block HCV entry at 1 μM and 5 μM while compounds 2 and 5 could block HCV entry at a higher concentration of 5 μM. Fig. 3B shows the results of the test for the VSVG-pp assay that indicated the specificity of HCV entry blocking. The specificity of anti-CD81 was taken as the positive control. Compound 2 showed the best specificity for HCV entry at 1 μM and 5 μM. The specificity for anti-HCV activity of compound 3 was slightly higher than that of the control and substrate at both concentrations. Candidates with both high HCV entry blocking activity and high specificity were desirable. Therefore, product 3 showed the best anti-HCV activity among the five compounds at concentrations of 1 μM and 5 μM. Product 2 showed better activity for blocking HCV entry at 5 μM (Fig. 3).

### Possible transformation pathway of UA by *G. roseum* CGMCC 3.3657

**Fig. 4:**
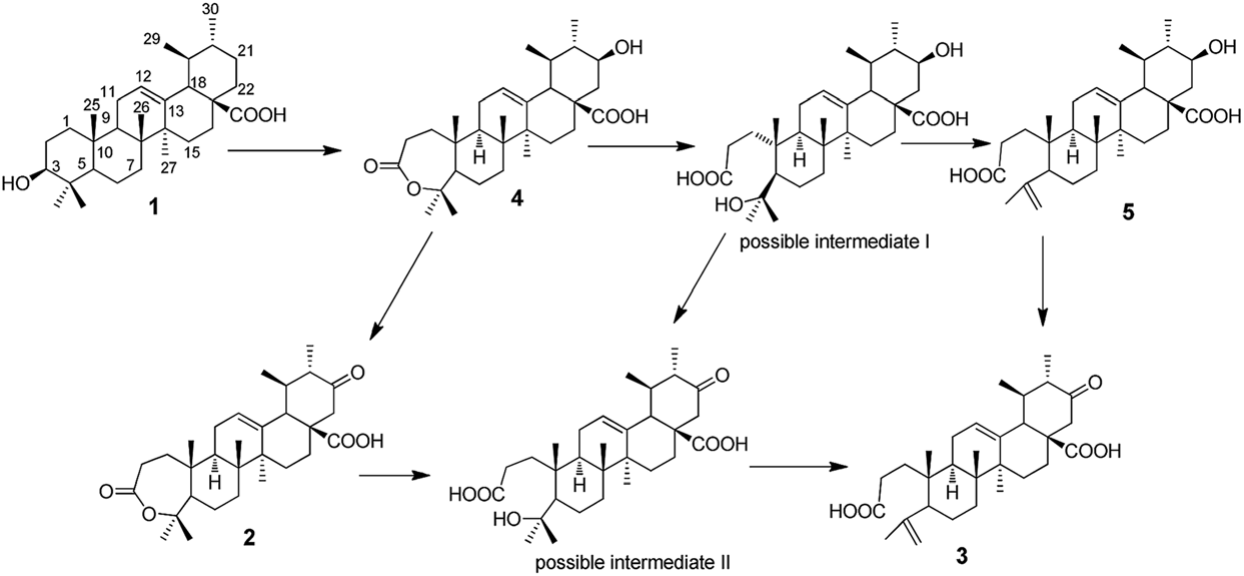
The possible transformation pathway of UA by *G. roseum* CGMCC 3.3657.

Based on the products obtained, the possible transformation pathway of UA by *G. roseum* CGMCC 3.3657 was proposed as shown in Fig. 4. The substrate 1 was first hydroxylated at C-21 to afford product 4. Product 4 was then either oxidised at C-21 to afford product 2 or its seven-membered ring was cleaved to form the possible intermediate I. Compound 5 was formed from possible intermediate I by the loss of one molecule of water. Compound 3 could be formed either from compound 5 or from compound 2 through the possible intermediate II as shown in Fig. 4.

## Experimental

### General experimental procedure

Crystallization was performed in MeOH and petroleum ether/THF. NMR spectra were recorded on a Bruker DRX-600 spectrometer operating at 600 MHz and 150 MHz in CDCl_3_ or DMSO-d_6_ with tetra-methylsilane (TMS) as an internal standard. High-resolution electrospray ionization mass spectra (ESI-MS) were obtained using a Thermo LTQ Orbitrap XL mass spectrometer. Standard pulse sequences were utilized to obtain HMQC and HMBC data. Column chromatography was carried out on silica gel (100–200 mesh and 300–400 mesh, Qingdao Oceanic Chemicals, China). TLC analyses were carried out on pre-coated silica gel GF254 plates (0.25 mm thick, Qingdao Oceanic Chemicals, China). Visualization of the TLC plates was performed by using a 10% H_2_SO_4_ in 95% ethanol spray reagent, followed by heating. General solvents and reagents were purchased from the Beijing Chemical Industry Company, Beijing, China. The microbes were purchased from the China General Microbiological Culture Collection centre (CGMCC). Optional rotations were recorded on a Perkin-Elmer341 at 509 nm and 20 °C. The melting point was recorded on the apparatus uncorrected.

### Substrate

The substrate ursolic acid (purity ≧98%) was purchased from Changsha Staherb Natural Ingredients Co. Ltd in China. Its structure was characterized by comparison of the ^1^H-NMR and ^13^C-NMR spectra measured with those reported in literature.^20,22^

### Microorganisms and culture conditions

The microorganism *G. roseum* CGMCC 3.3657 was purchased from the China General Microbiological Culture Collection centre (CGMCC). The preliminary screening experiment of *G. roseum* CGMCC 3.3657 was carried out in potato dextrose medium (consisting of 200 g potato boiled for 20 min; dextrose, 20 g; peptone, 10 g; distilled H_2_O, 1000 mL). Standard two-stage fermentation was applied. The cultures were grown in 100 mL Erlenmeyer flasks containing 40 mL of medium. After incubation at 28 °C and 160 rpm in the shaker for 3 days, the substrate, 0.5 mg, (dissolved in ethanol) was added into the flasks for another 20 days and harvested and extracted three times with equal volumes of EtOAc. The extracts were evaporated under reduced pressure on a rotary evaporator (50 °C) and detected by TLC. Two culture controls with only the substrate and only the fungi, respectively, were used simultaneously under the same conditions as above.

### Biotransformation procedures and isolation of metabolites

The preparative scale biotransformation of UA by *G. roseum* CGMCC 3.3657 was carried out in ten 1000 mL flasks each containing 400 mL of potato dextrose medium. The cultures were incubated for 72 hours before 400 mg of substrate in total (suspended in ethanol) was evenly added. The conditions and procedures for incubation and extraction were the same as described above. The crude extract, 740 mg, was subjected to column chromatography on silica gel (300–400 mesh, 25 g) with stepwise elution using petroleum ether/acetone/acetic acid from 100 : 1 : 0.1 to acetone. Four fractions were obtained: fraction A (36 mg), fraction B (38 mg), fraction C (194 mg) and fraction D (70 mg).

Fraction A was further re-crystallized in petroleum ether/THF to afford metabolite 2 (25 mg, 6.25%). Fraction B was further purified by column chromatography on silica gel (300–400 mesh, 7 g) by eluting stepwise with CHCl_3_/petroleum ether (85 : 35) to afford metabolite 3 (4 mg, 1%). Fraction C was further re-crystallized in methanol to afford metabolite 4 (32 mg, 8%) and the mother liquor was further purified by column chromatography on silica gel (300–400 mesh, 9.5 g) by eluting stepwise with CHCl_3_/MeOH from 100 : 2 to 100 : 8 and with petroleum ether/acetone from 100 : 11 to 11 : 13 to afford metabolite 5 (6 mg, 1.5%).

Metabolite 2: white crystal, mp 263–265 °C, [*α*]^D^_20_ +54.4° (*c* = 1.23 × 10^−4^, EtOH), HR-ESI-MS: 483.3104 [M−H]^−^ (calcd 483.3105). For ^13^C-NMR (150 MHz, DMSO-d_6_) and ^1^H-NMR (600 MHz, DMSO-d_6_) spectra see Tables 1 and 2.

**Table 1:** ^13^C-NMR data of compounds 2–5 (150 MHz)^a^

No.	Product 2	Product 3	Product 4	Product 5
1	37.8	34.1	38.3	33.9
2	31.7	28.5	32.1	28.2
3	174.2	178.5	175.2	174.1
4	85.4	147.1	86.2	147.1
5	53.4	51.0	54.7	49.3
6	25.1	24.5	23.7	24.0
7	31.7	31.8	32.4	31.3
8	39.1	39.6	39.6	38.7
9	46.5	38.2	47.4	37.1
10	39.2	39.5	39.8	38.7
11	23.3	24.0	25.2	23.0
12	126.0	127.6	126.4	124.8
13	137.2	137.1	137.5	138.1
14	41.6	42.5	42.2	42.1
15	27.4	28.1	28.0	27.6
16	25.6	26.0	22.9	24.8
17	50.1	51.4	48.7	47.8
18	51.7	52.7	52.2	52.2
19	40.6	41.8	38.0	37.5
20	50.1	51.3	46.6	46.4
21	209.5	209.5	71.2	69.0
22	49.8	50.7	44.6	45.2
23	31.8	113.7	32.4	113.4
24	30.4	23.6	25.8	23.5
25	16.8	19.5	17.1	19.3
26	16.6	17.3	16.9	17.0
27	23.2	23.9	23.3	23.0
28	175.7	179.5	179.6	177.4
29	18.1	18.7	17.1	17.3
30	12.5	12.6	15.6	15.8

a Solvent DMSO-d_6_ was used for products 2 and 5; solvent CDCl_3_ was used for products 3 and 4.

**Table 2:** The characteristic ^1^H-NMR data of compounds 2–5 (600 MHz)^a^

No.	Product 2	Product 3	Product 4	Product 5
1	1.61 (o), 1.49 (o)	1.61 (m), 1.58 (m)	1.56 (m), 1.79 (m)	1.45 (m)
2	2.58 (o)	2.39 (m), 2.56 (m)	2.61 (m)	2.05 (m)
3	—	—	—	—
4	—	—	—	—
5	1.68 (o)	1.92 (o)	1.66 (d, 10.8)	1.96 (dd, 1.8, 12.6)
6	1.76 (m)	1.37 (m)	1.99 (m)	1.30 (m)
7	1.48 (m), 1.24 (m)	1.47 (m), 1.26 (m)	1.36 (m)	1.21 (m), 1.47 (m)
8	—	—	—	—
9	1.61 (o)	1.68 (m)	1.62 (m)	1.86 (m)
10	—	—	—	—
11	1.94 (o)	1.93 (m)	1.85 (m)	1.04 (o)
12	5.32 (t, 3.6)	5.40 (t, 3.6)	5.32 (t-like)	5.16 (t, 3.6)
13	—	—	—	—
14	—	—	—	—
15	1.77 (o), 1.03 (o)	1.11 (m)	1.11 (m), 1.84 (m)	1.75 (m)
16	1.67 (m), 1.48 (m)	1.85 (m), 1.59 (m)	1.52 (m)	1.65 (m)
17	—	—	—	—
18	2.64 (d, 10.8)	2.68 (d, 11.4)	2.249 (d, 11.4)	2.11 (d, 10.8)
19	1.69 (o)	1.77 (m)	1.44 (m)	1.40 (m)
20	2.23 (dd, 6.0, 10.8)	2.14 (dd, 6.0, 10.8)	0.98 (o)	0.79 (m)
21	—	—	3.43 (td, 4.2, 10.8)	3.19 (td, 4.2, 10.8)
22	2.14 (d, 13.2), 2.69 (d, 12.6)	2.65 (d, 12.6), 2.39 (d, 12.6)	1.57 (m), 2.41 (dd, 4.2, 12.6)	1.80 (m), 1.35 (m)
23	1.40 (s)	4.86 (brs), 4.65 (brs)	1.48 (s)	4.65 (brs), 4.84 (brs)
24	1.34 (s)	1.72 (s)	1.42 (s)	1.71 (s)
25	1.08 (s)	0.92 (s)	1.15 (s)	0.86 (s)
26	0.81 (s)	0.82 (s)	0.84 (s)	0.80 (s)
27	1.02 (s)	1.05 (o)	1.07 (s)	1.05 (s)
28	—	—	—	—
29	0.96 (d, 6.6)	1.01 (d, 6.6)	0.91 (d, 6.0)	0.84 (d, 6.6)
30	0.93 (d, 6.6)	1.05 (d, 6.6)	1.08 (d, 6.0)	0.98 (d, 6.6)

a Mult, multiplicity: s, singlet; d, doublet; t, triplet; o, overlap; solvent DMSO-d_6_ was used for products 2 and 5; solvent CDCl_3_ was used for products 3 and 4.

Metabolite 3: white crystal, mp 217–220 °C, [*α*]^D^_20_ +27.2° (*c* = 8.46 × 10^−3^, EtOH), HR-ESI-MS: 483.3105 [M−H]^−^ (calcd 483.3105). For ^13^C-NMR (150 MHz, CDCl_3_) and ^1^H-NMR (600 MHz, CDCl_3_) spectra see Tables 1 and 2.

Metabolite 4: white solid, mp 204–206 °C, [*α*]^D^_20_ +63.6 (*c* = 6.92 × 10^−3^, EtOH), HR-ESI-MS: 485.3263 [M−H]^−^ (calcd 485.3262). For ^13^C-NMR (150 MHz, CDCl_3_) and ^1^H-NMR (600 MHz, CDCl_3_) spectra see Tables 1 and 2.

Metabolite 5: white solid, mp 201–203 °C, [*α*]^D^_20_ +80.4° (*c* = 2.07 × 10^−4^, EtOH), HR-ESI-MS: 485.3215 [M−H]^−^ (calcd 485.3262). For ^13^C-NMR (150 MHz, DMSO-d_6_) and ^1^H-NMR (600 MHz, DMSO-d_6_) spectra see Tables 1 and 2.

### Anti-HCV activity of UA and biotransformation product evaluation

It was reported that the connection of the virus to the cell surface after viral entry was the first step in a cascade of interactions. Host cell infection is initiated by binding of the virion to cell-surface receptors. Using recombinant HCV envelope glycoproteins and a HCV-pseudo type particle (HCVpp), several cell surface molecules have been identified as interacting with the HCV during viral binding and entry. Currently, three host proteins, CD81, SR-BI and claudin-1 (CLDN1), are thought to be essential (co-)receptors for HCV entry. CD81 was regarded as the best studied of the HCV (co-)receptors. At present, treatments for HCV infection are limited and vaccines to prevent HCV infection are not available. Studying interactions with HCV entry seems to be a promising approach for drug design and screening. The anti-HCV activity was evaluated by the method described by Hengli Tang.^23^ The substrate and transformation products were tested for their capacity to block HCV entry by (HCVpp) entry assay. The specificity of anti-HCV entry was evaluated by using a vesicular stomatitis virus G protein pseudo-particle (VSV G-pp).

## Conclusions

In recent years, the idea of “returning to nature” has made researchers begin to look back to natural medicine. Natural products are structurally and biologically interesting compounds. Isolated natural compounds are often available in minute amounts. Thus, the synthesis of natural products also provides a powerful means to solve supply problems in clinical trials and market the products in bulk amounts. Biotransformation was regarded as an alternative to produce new compounds. In this paper it is reported that four new products from UA were afforded by microbial transformation. A Baeyer–Villiger oxidative reaction occurred which was catalyzed by *G. roseum* CGMCC 3.3657. A transformation pathway was proposed. Moreover, the anti-HCV activity of UA and the derived products was tested. Compounds 2 and 3 showed a small improvement in activity compared to the substrate. The results reported in this article will help future researchers for further investigation.

## Conflicts of interest

There are no conflicts to declare.

## Supplementary Material

RA-008-C8RA01217B-s001
