# Supplementary material for: Biocatalysis of ursolic acid by the fungus Gliocladium roseum CGMCC 3.3657 and resulting anti-HCV activity
Source: RSC Adv. 2018 May 3;8(29):16400–5. doi: 10.1039/c8ra01217b (PMC9080225; doi:10.1039/c8ra01217b)
Supplement: RA-008-C8RA01217B-s001 [file RA-008-C8RA01217B-s001.pdf]

Electronic Supplementary Material (ESI) for RSC Advances.
This journal is © The Royal Society of Chemistry 2018

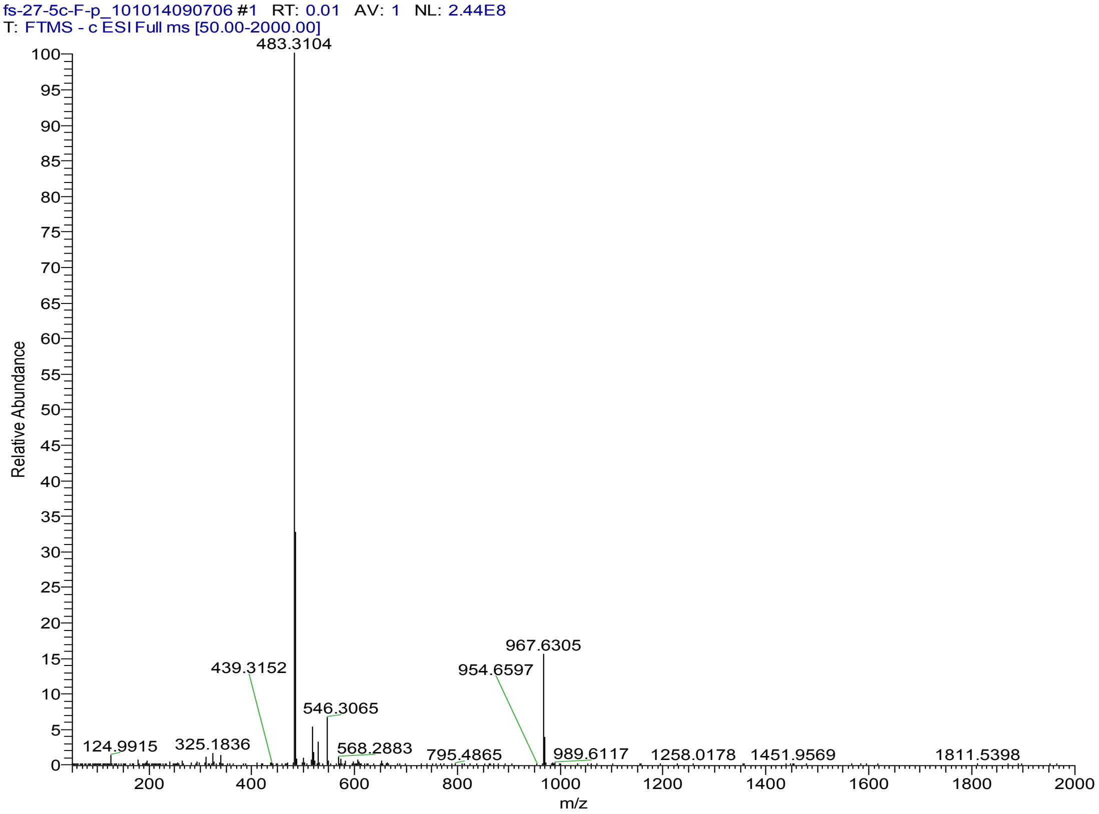

Appendix 2-1. Mass spectrum of compound **2.**

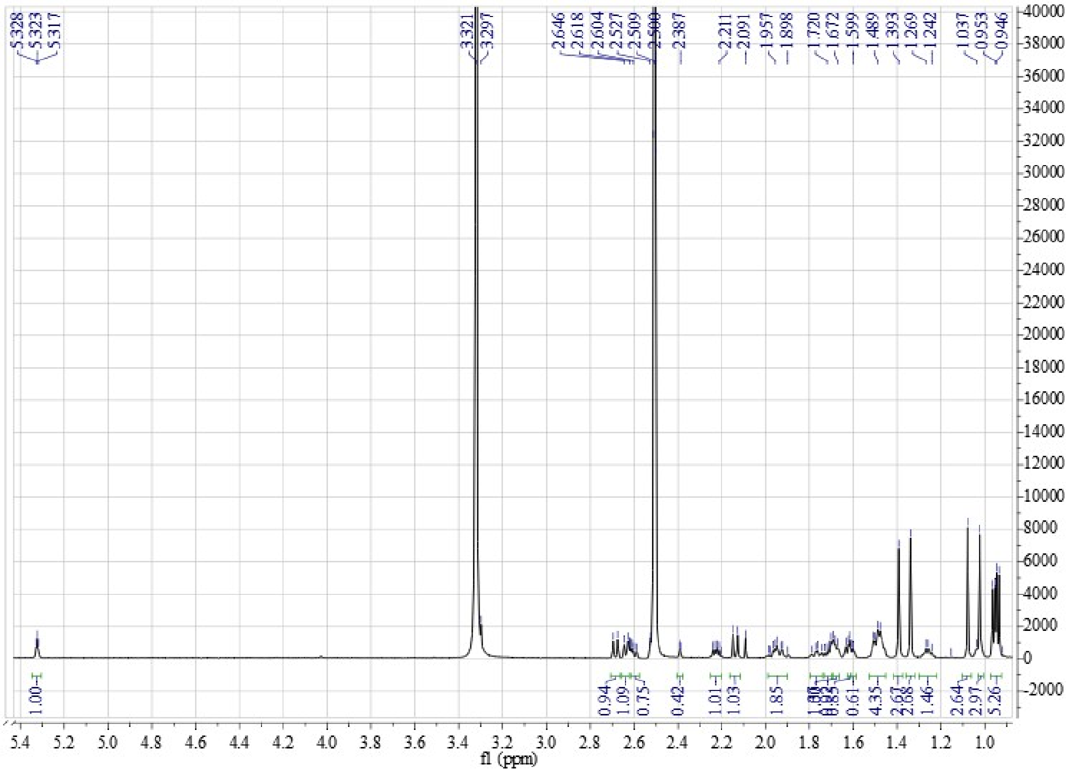

Appendix 2-2. ^{1}H-NMR spectrum of compound **2.**

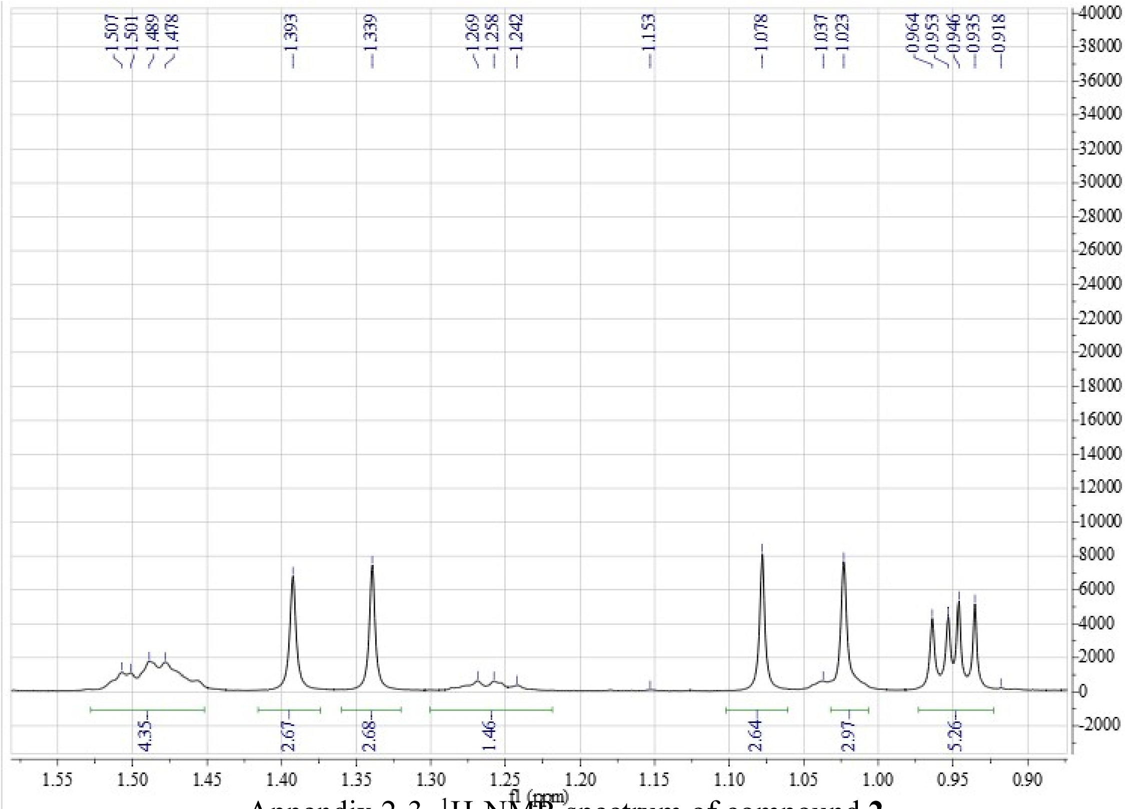

Appendix 2-3. ^{1}H-NMR spectrum of compound **2.**

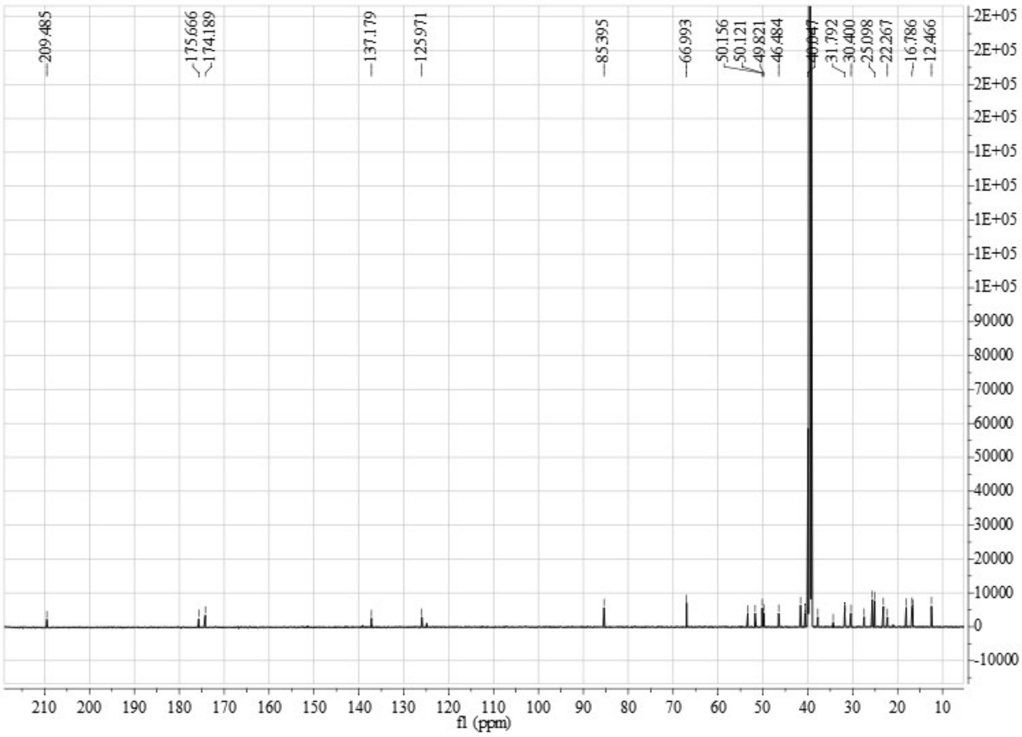

Appendix 2-4. ^{13}C-NMR spectrum of compound **2.**

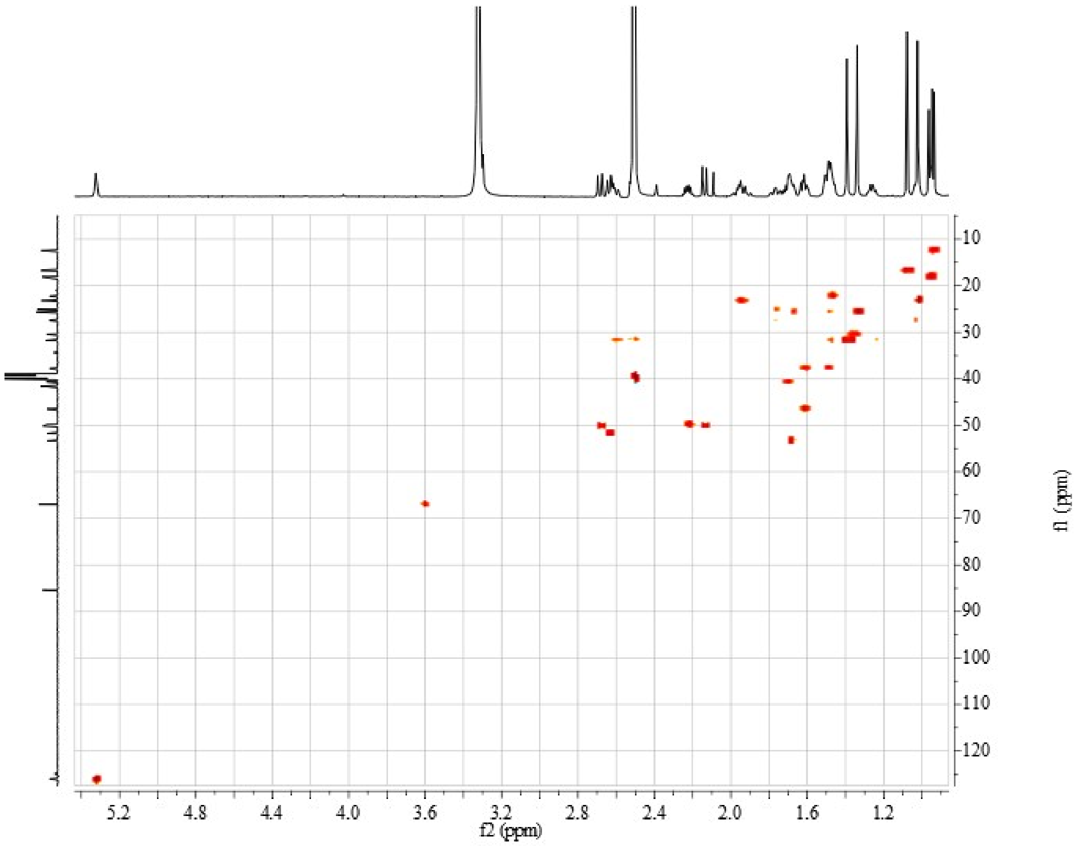

Appendix 2-5. HMQC spectrum of compound **2.**

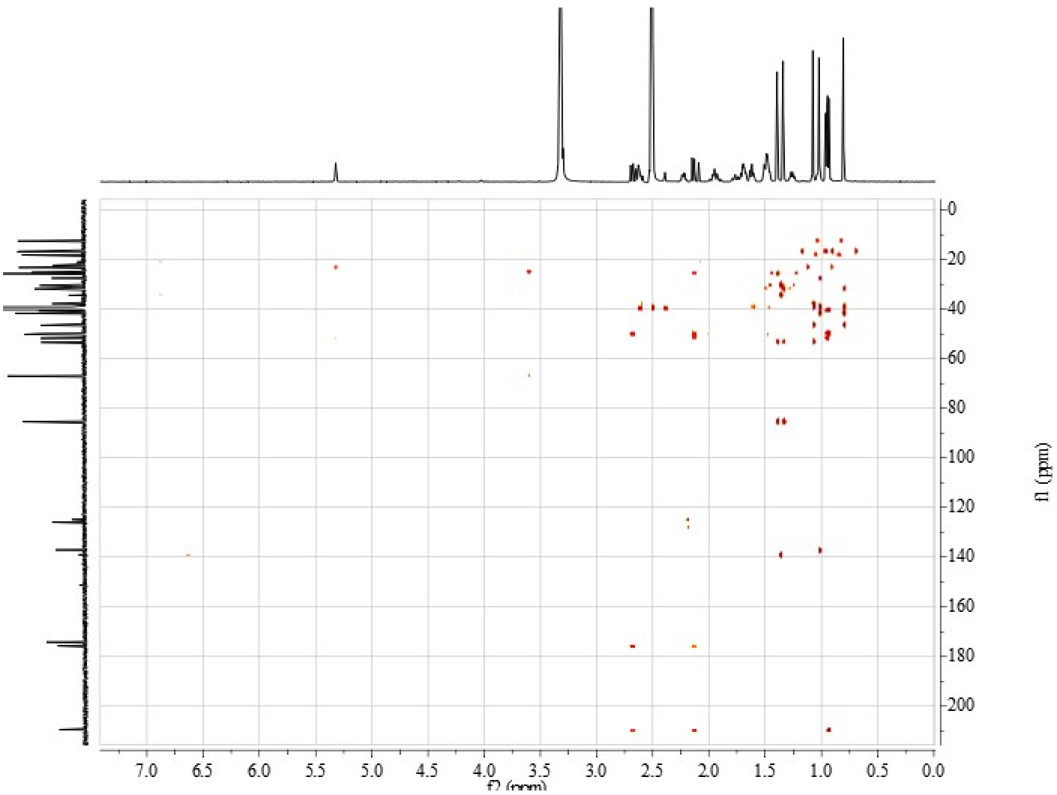

Appendix 2-6. HMBC spectrum of compound **2.**

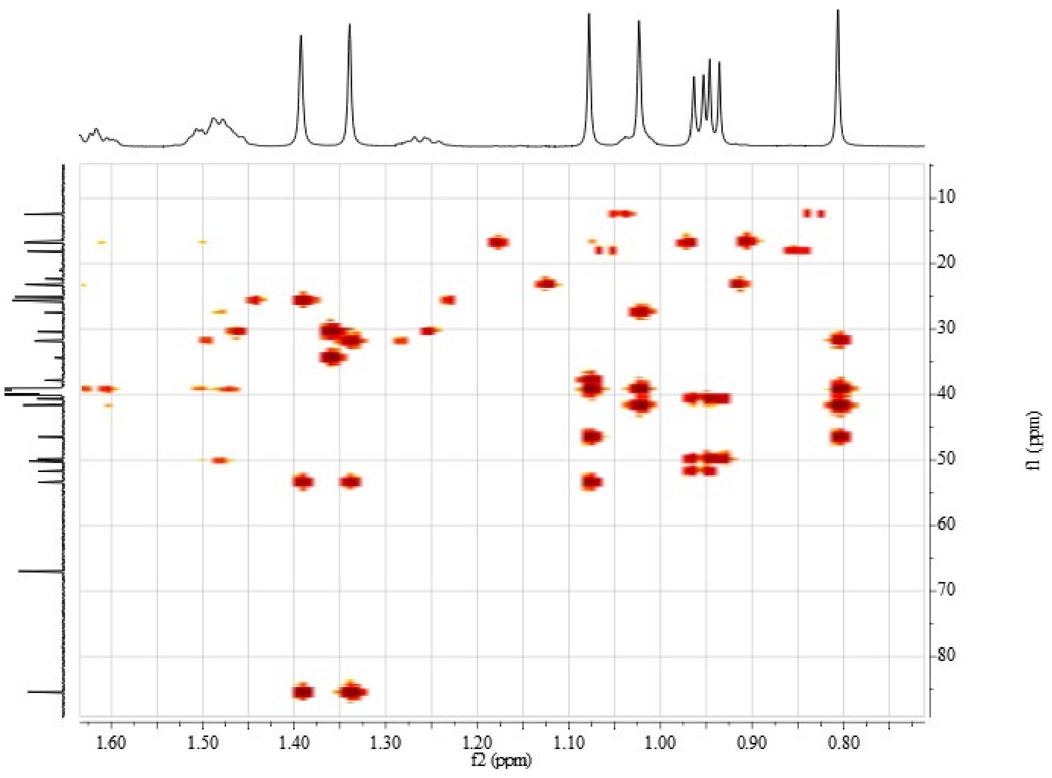

Appendix 2-7. HMBC spectrum of compound **2.**

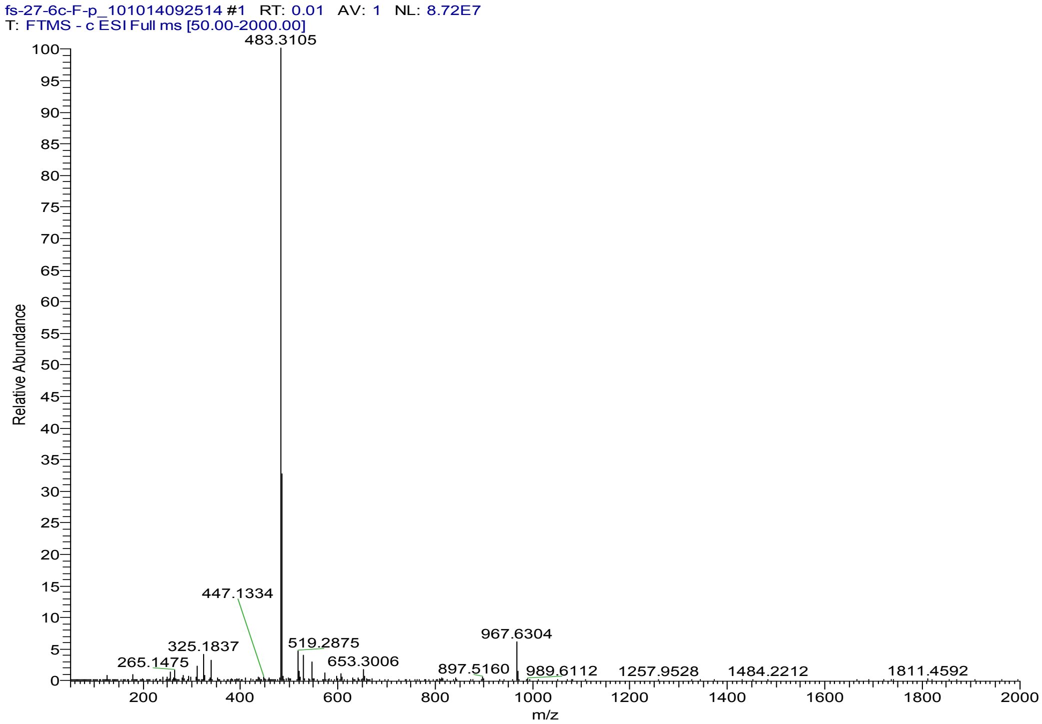

Appendix 3-1. Mass spectrum of compound **3.**

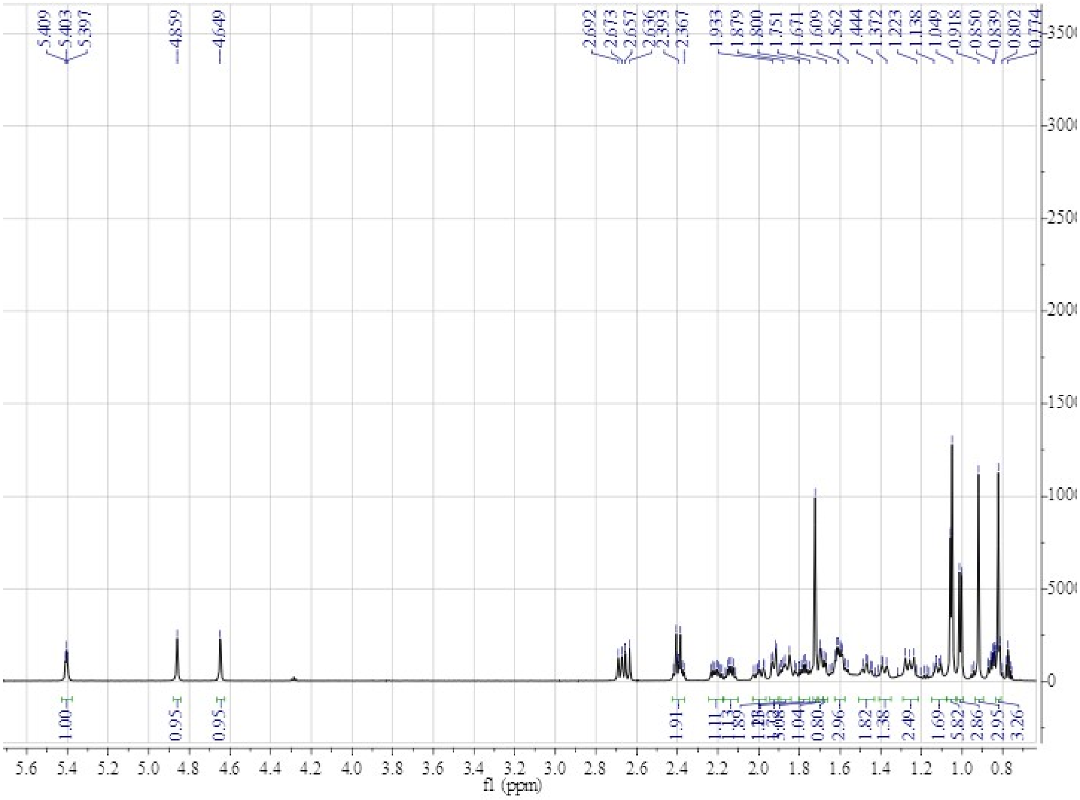

Appendix 3-2. ^{1}H-NMR spectrum of compound **3.**

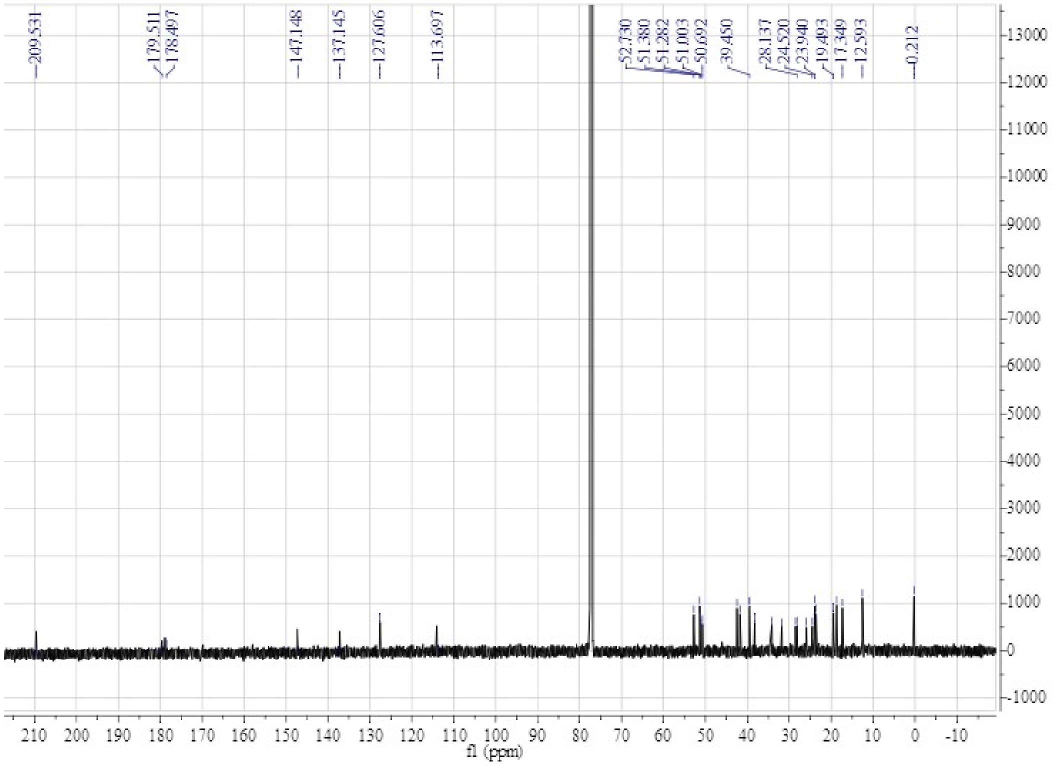

Appendix 3-3. ^{13}C-NMR spectrum of compound **3.**

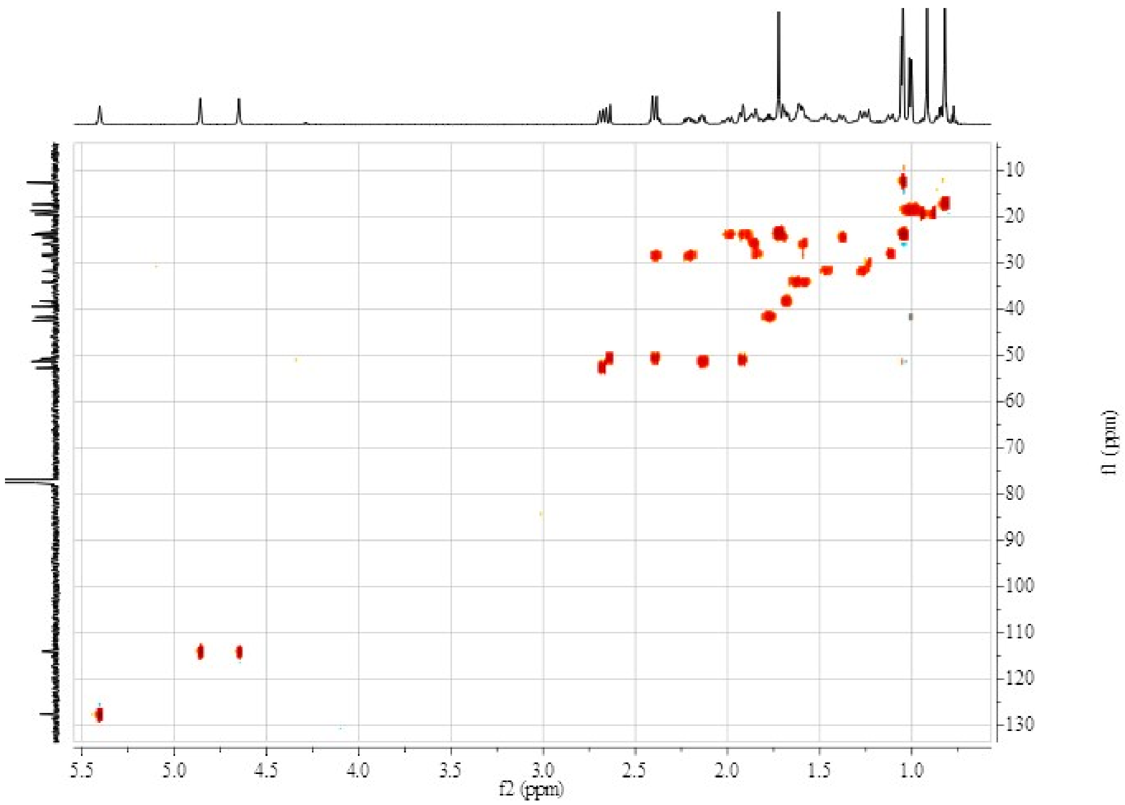

Appendix 3-4. HMQC spectrum of compound **3.**

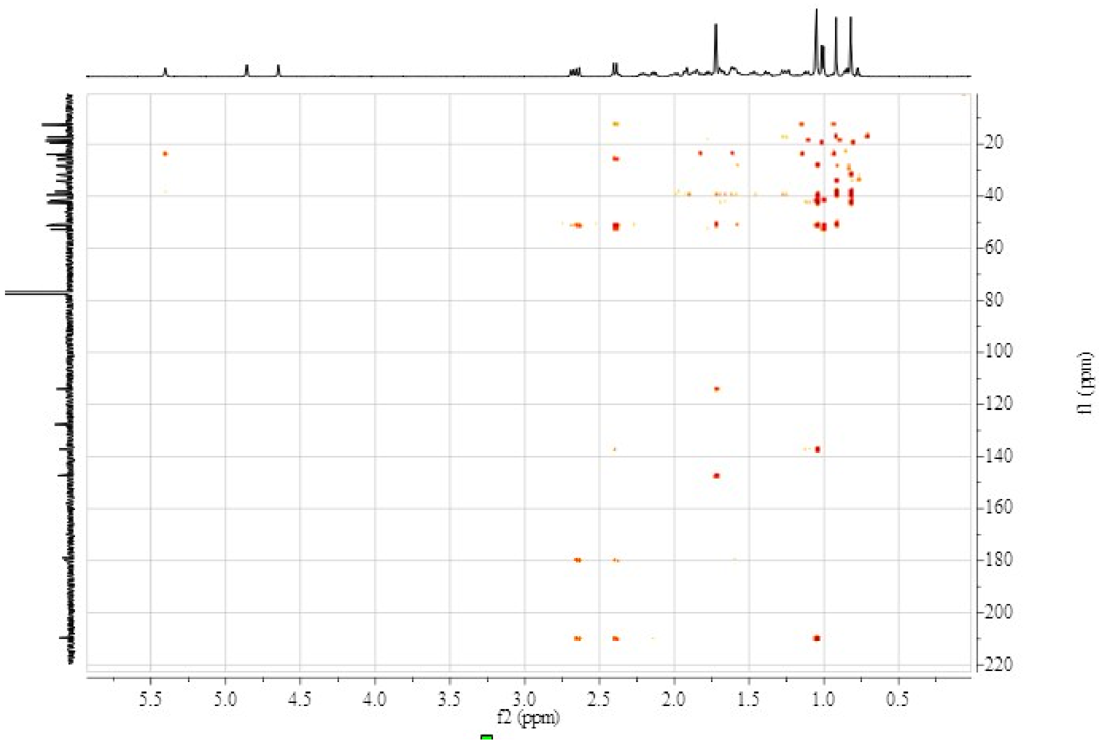

Appendix 3-5. HMBC spectrum of compound **3.**

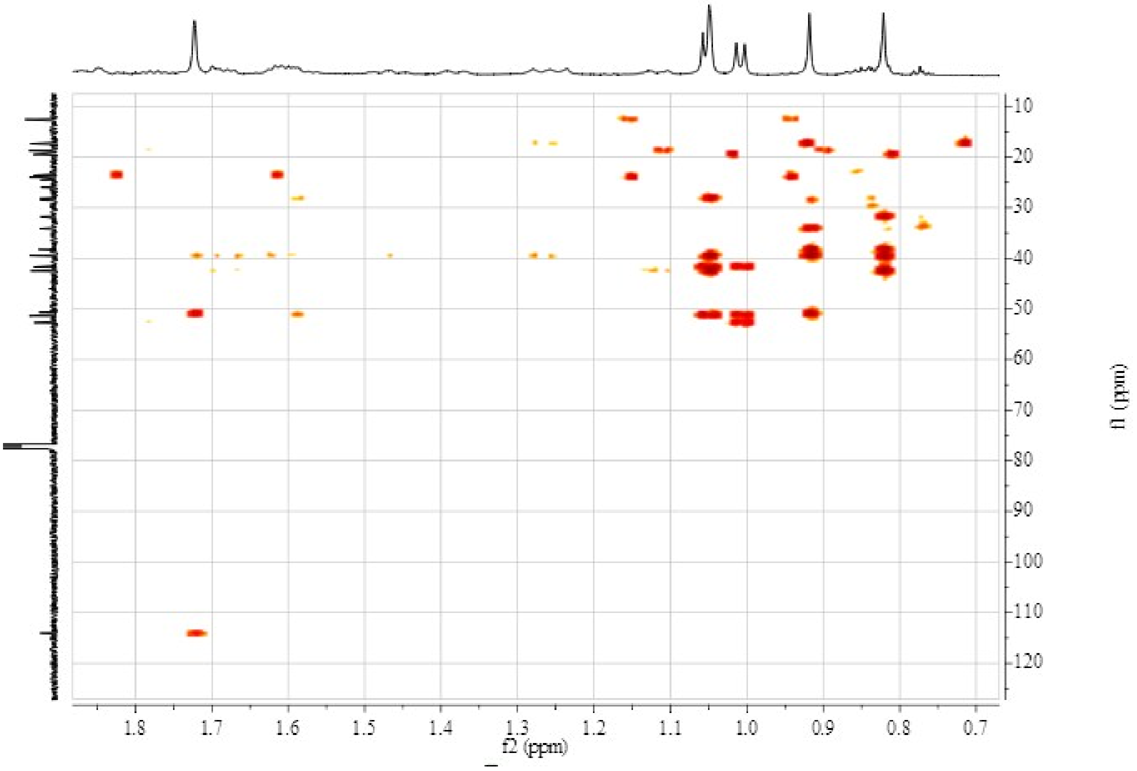

Appendix 3-6. HMBC spectrum of compound **3.**

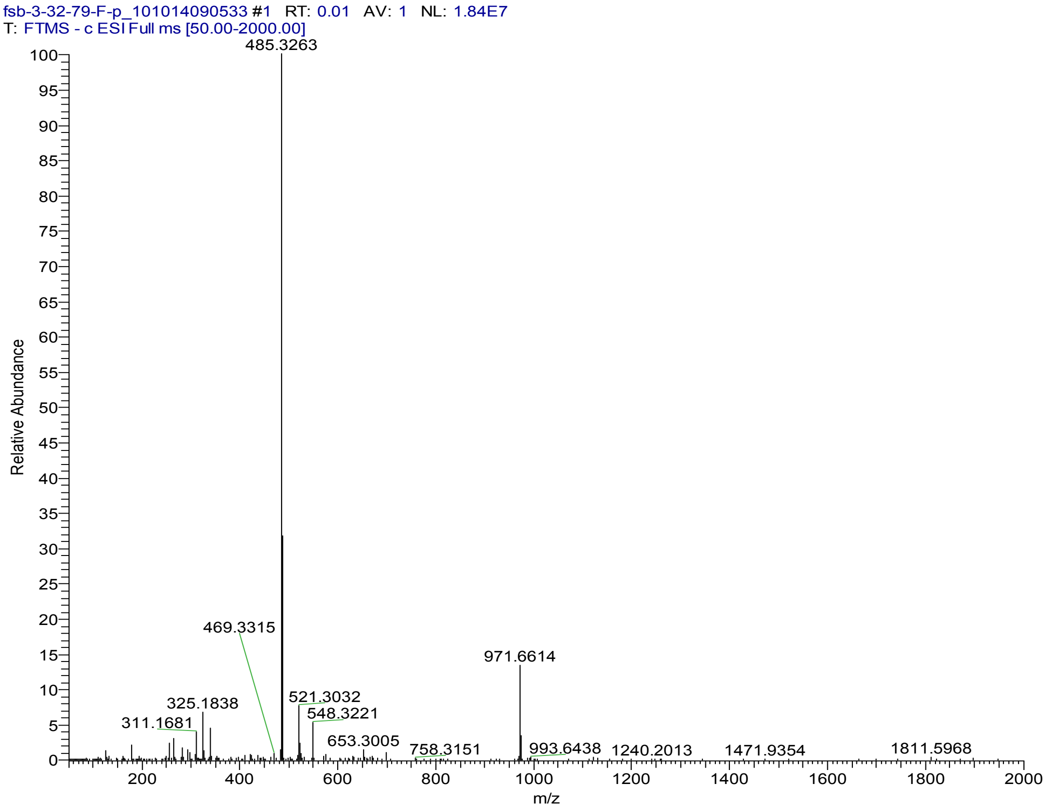

Appendix 4-1. Mass spectrum of compound **4.**

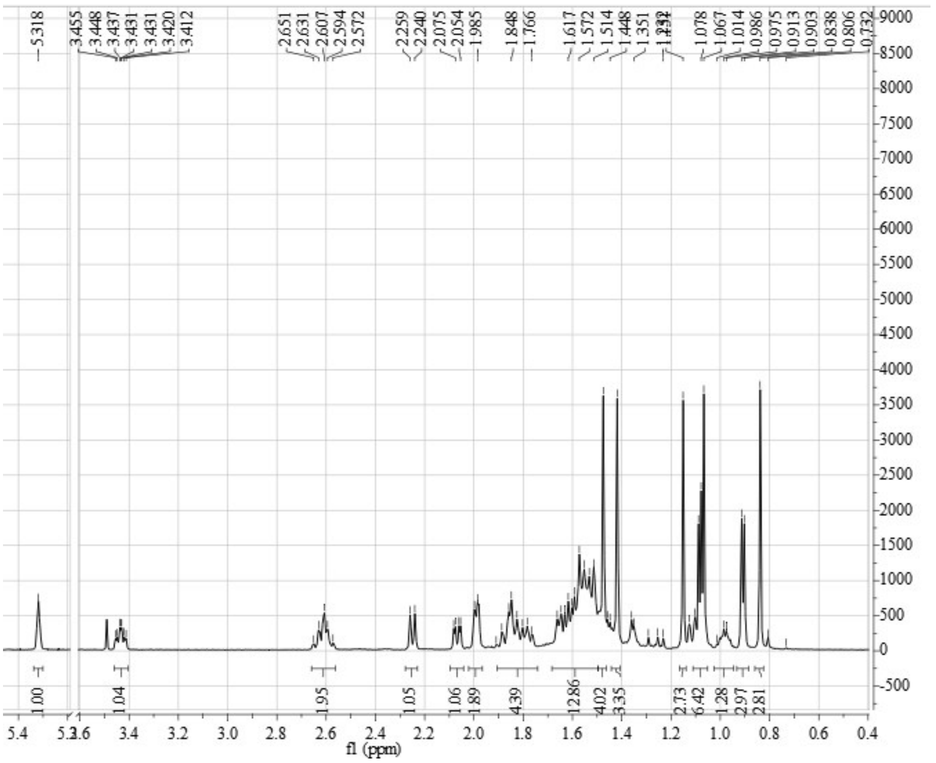

Appendix 4-2. ^{1}H-NMR spectrum of compound **4.**

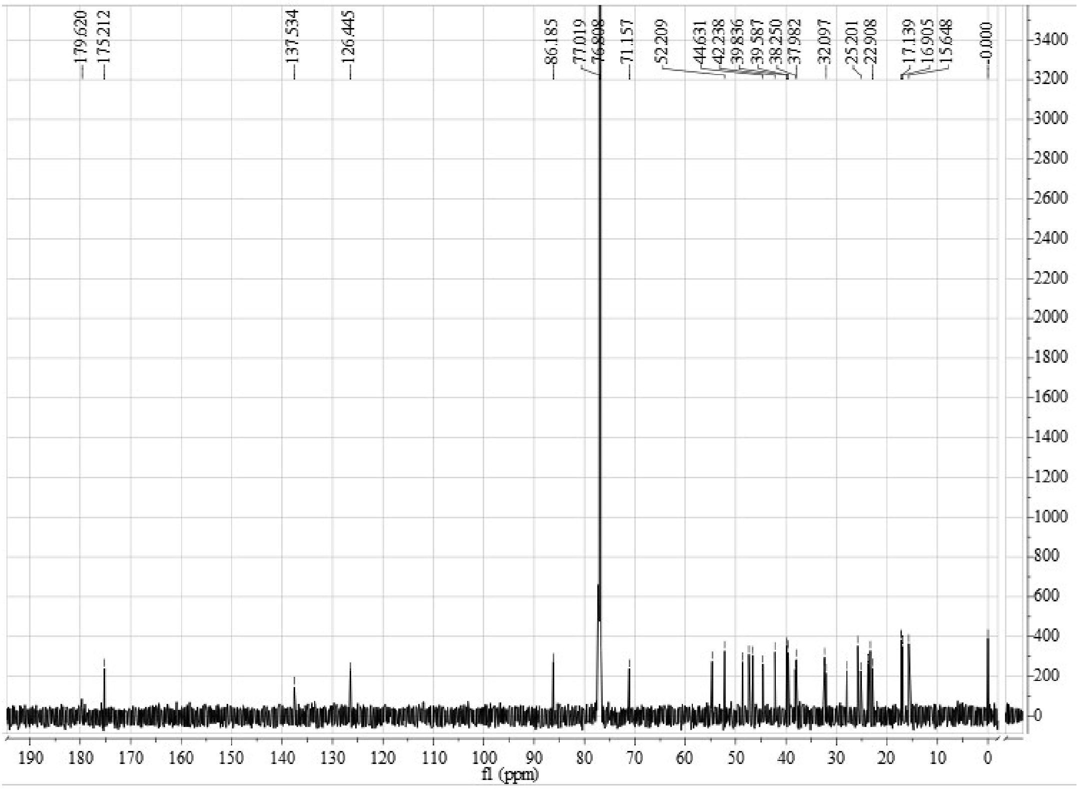

Appendix 4-3. ^{13}C-NMR spectrum of compound **4.**

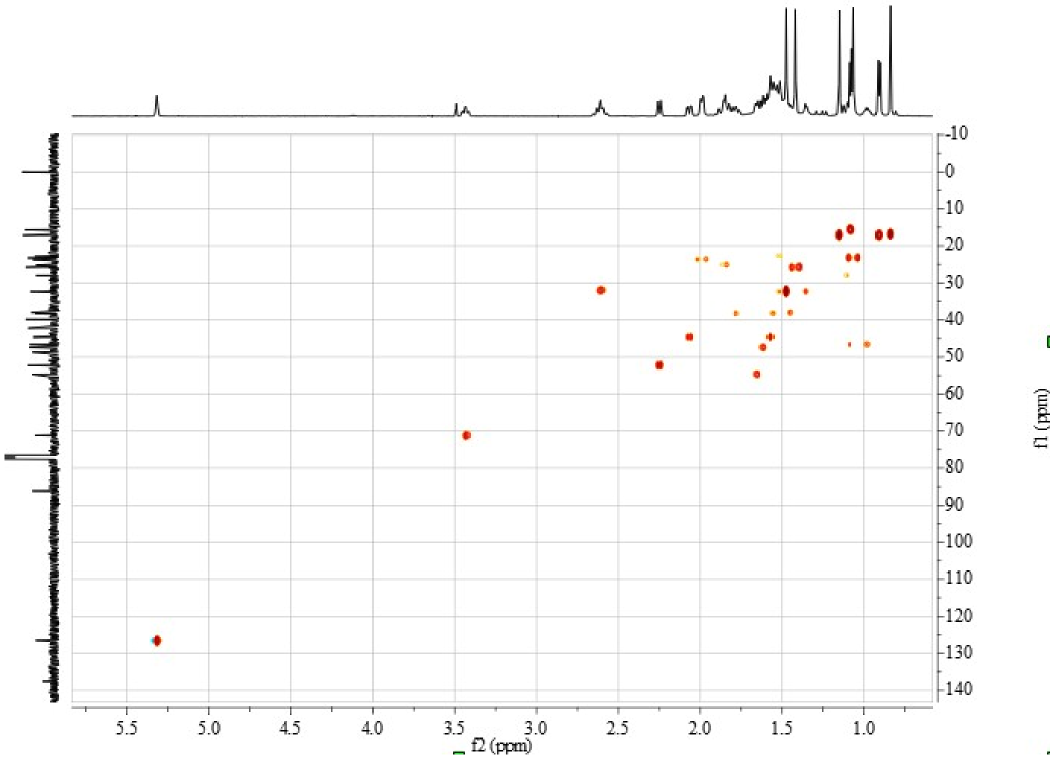

Appendix 4-4. HMQC spectrum of compound **4.**

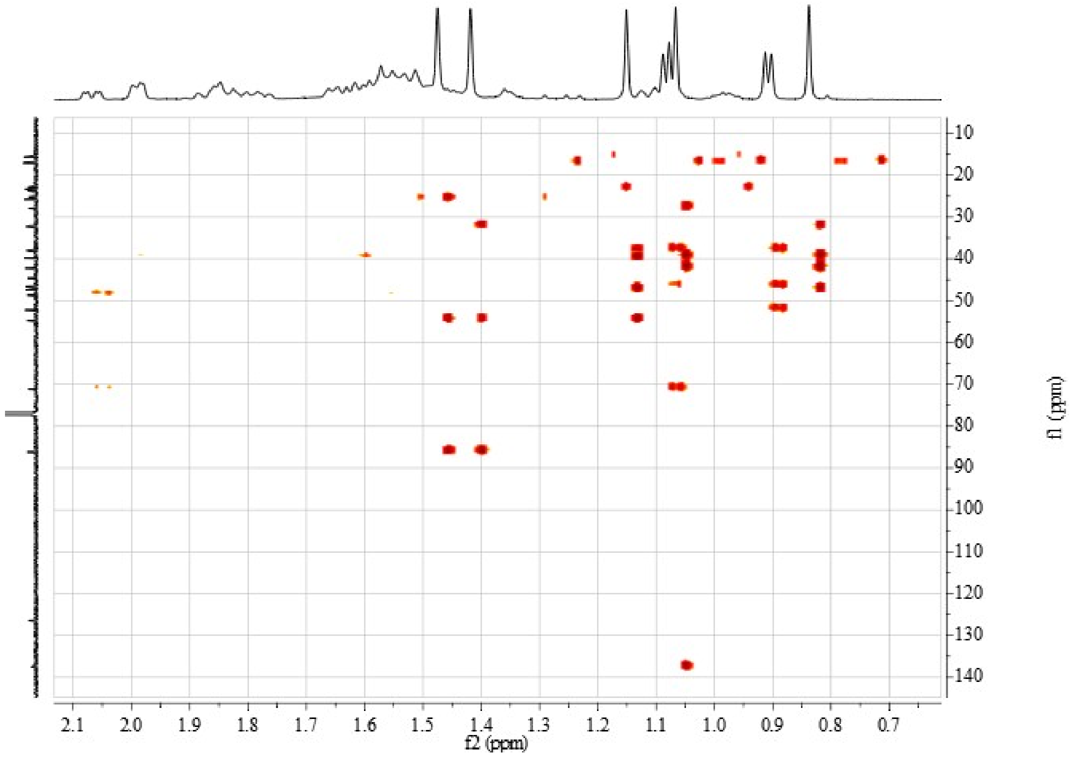

Appendix 4-5. HMBC spectrum of compound **4**.

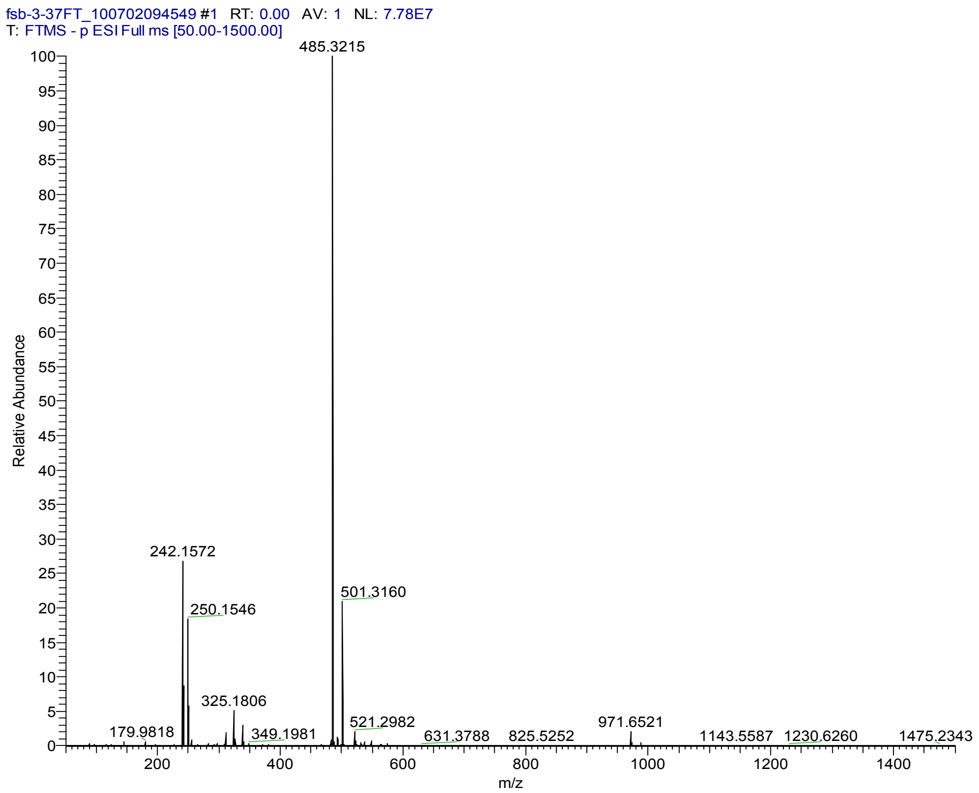

Appendix 5-1. Mass spectrum of compound **5.**

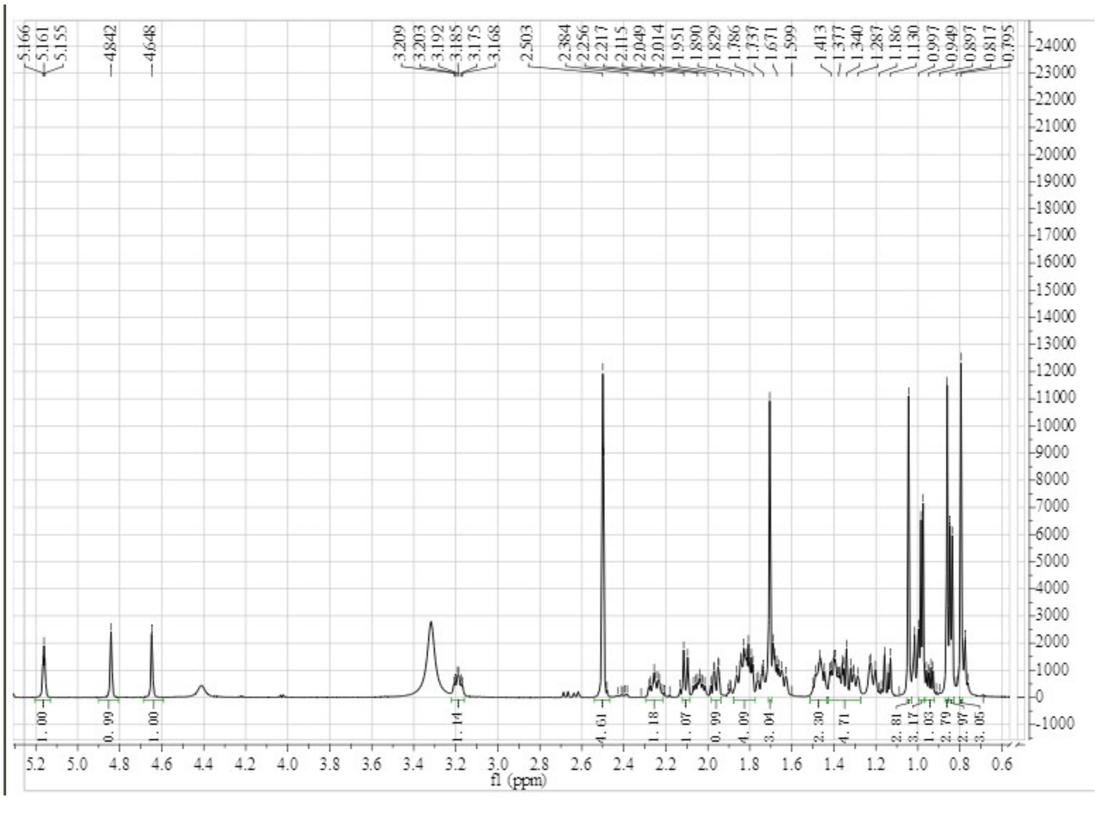

Appendix 5-2. ^{1}H-NMR spectrum of compound **5.**

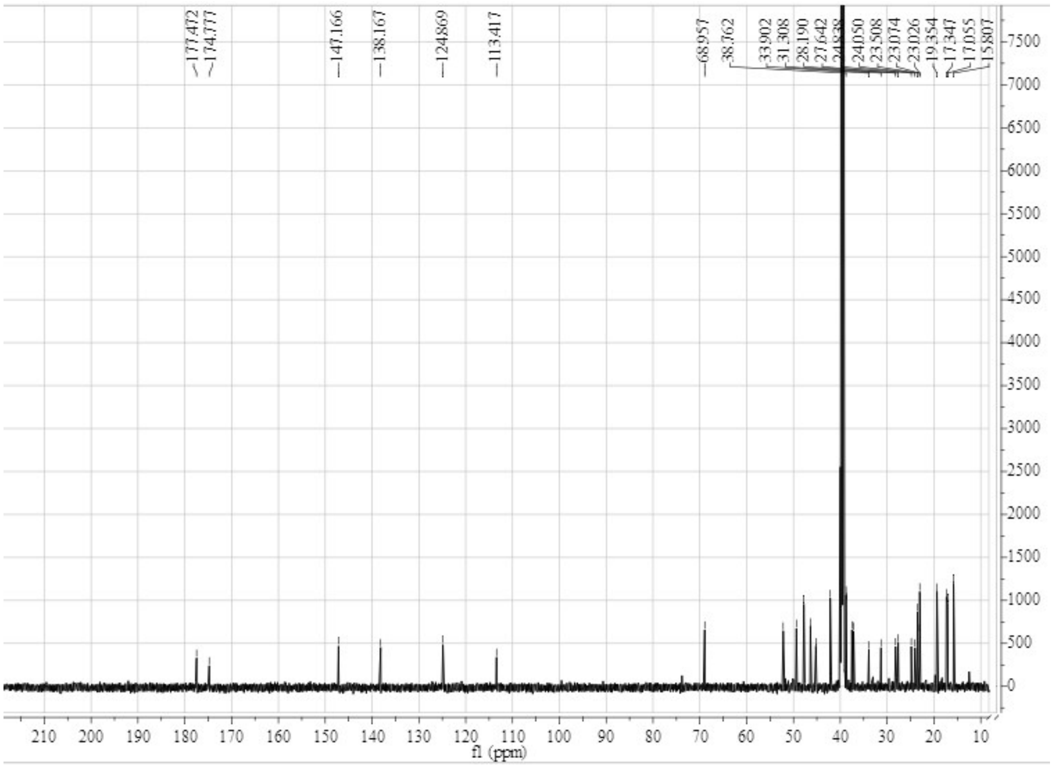

Appendix 5-3. ^{13}C-NMR spectrum of compound **5.**

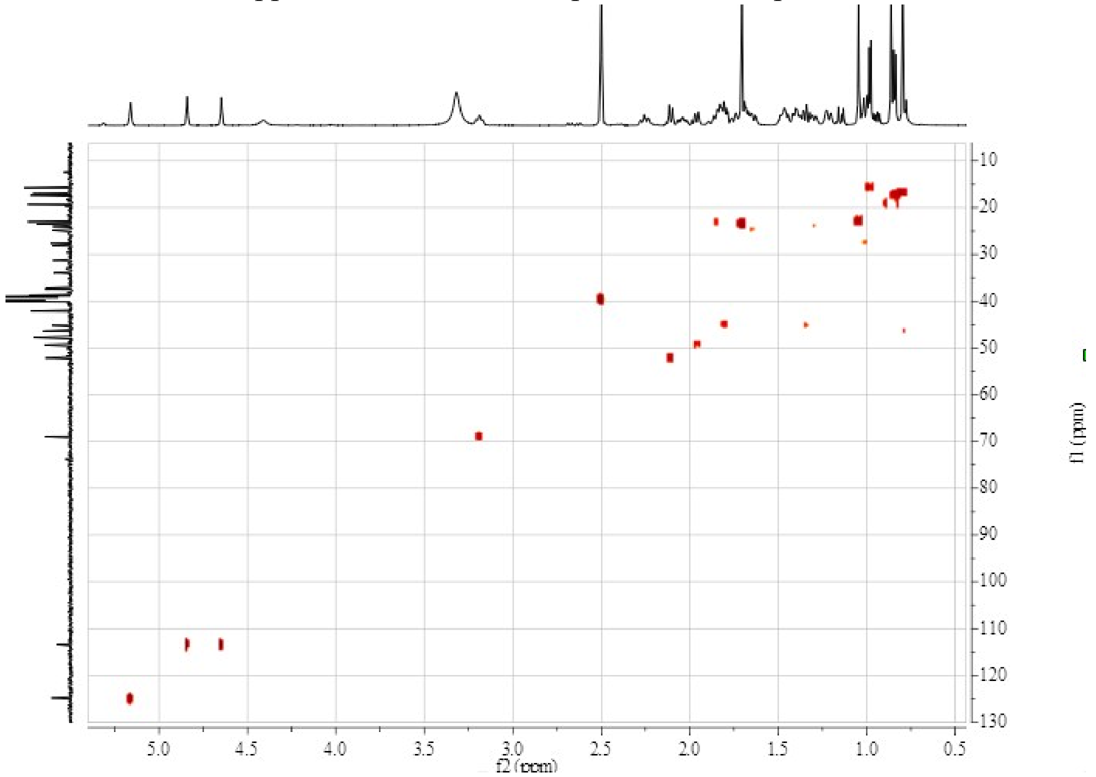

Appendix 5-4. HMQC spectrum of compound **5.**

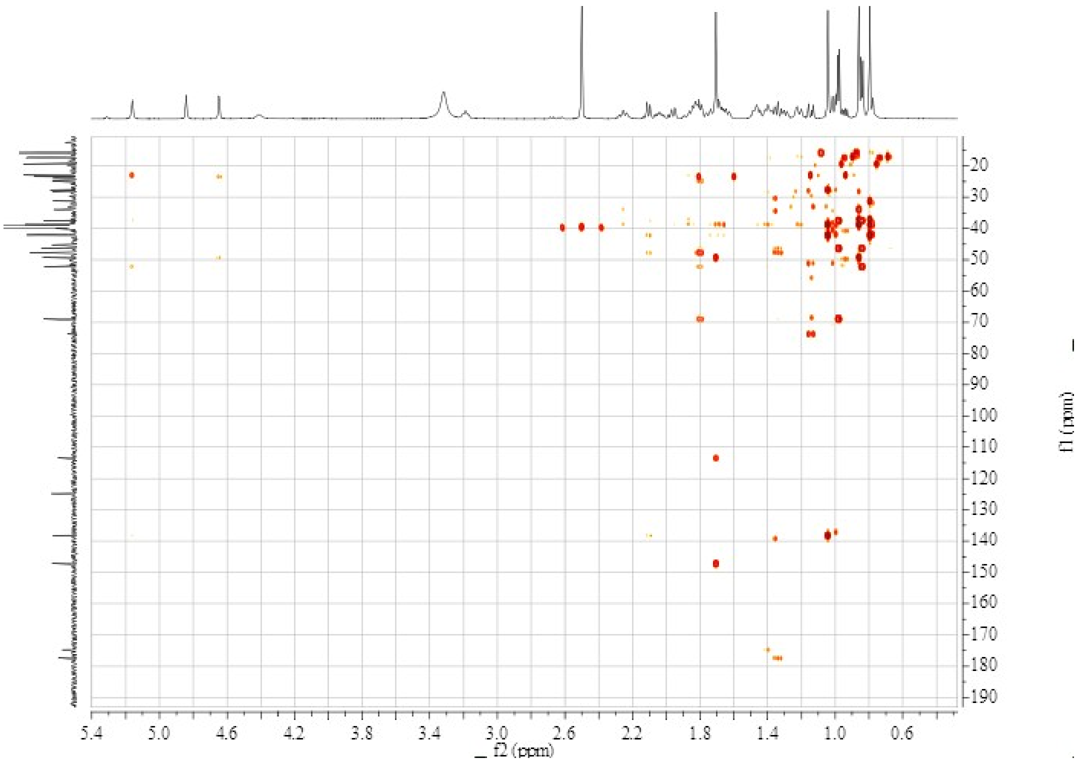

Appendix 5-5. HMBC spectrum of compound **5.**

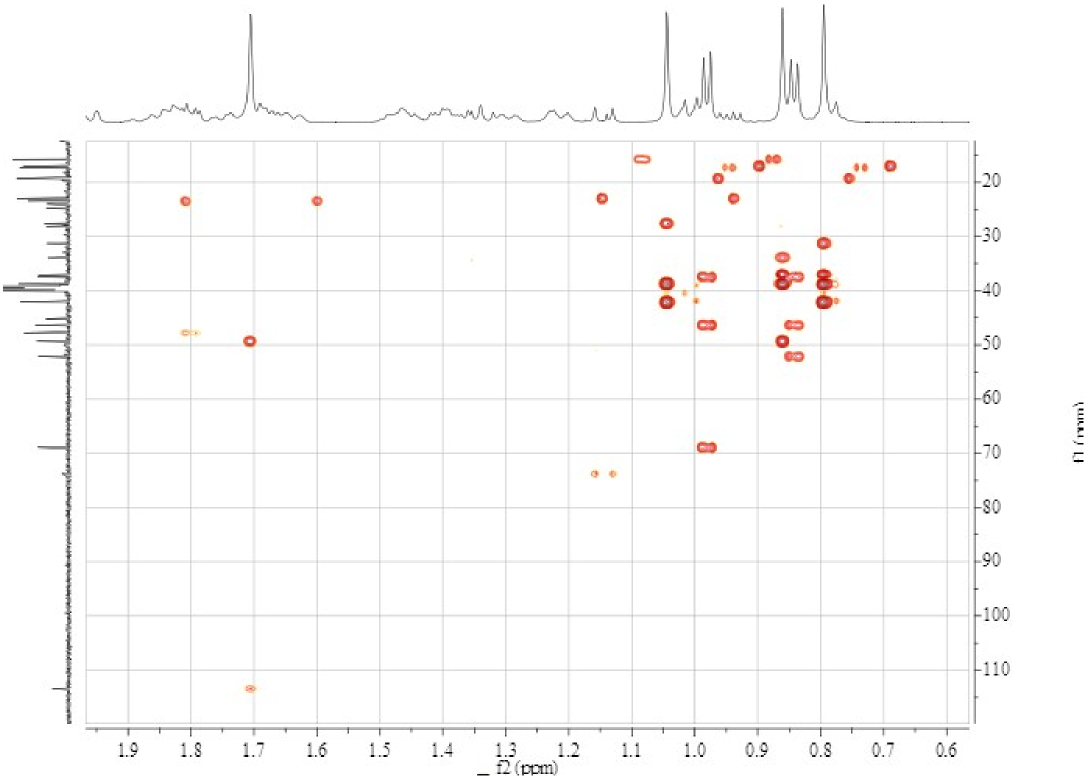

Appendix 5-6. HMBC spectrum of compound **5.**
